# Altered Expression of Hypoxia-Inducible Factor-1α (HIF-1α) and Its Regulatory Genes in Gastric Cancer Tissues

**DOI:** 10.1371/journal.pone.0099835

**Published:** 2014-06-13

**Authors:** Jihan Wang, Zhaohui Ni, Zipeng Duan, Guoqing Wang, Fan Li

**Affiliations:** 1 Department of Pathogenobiology, Jilin Key Laboratory of Biomedical Materials, College of Basic Medical Science, Jilin University, Changchun, China; 2 The Key Laboratory for Bionics Engineering, Ministry of Education, China, Jilin University, Changchun, China; University of Nebraska Medical Center, United States of America

## Abstract

Tissue hypoxia induces reprogramming of cell metabolism and may result in normal cell transformation and cancer progression. Hypoxia-inducible factor 1-alpha (HIF-1α), the key transcription factor, plays an important role in gastric cancer development and progression. This study aimed to investigate the underlying regulatory signaling pathway in gastric cancer using gastric cancer tissue specimens. The integration of gene expression profile and transcriptional regulatory element database (TRED) was pursued to identify HIF-1α ↔ NFκB1 → BRCA1 → STAT3 ← STAT1 gene pathways and their regulated genes. The data showed that there were 82 differentially expressed genes that could be regulated by these five transcription factors in gastric cancer tissues and these genes formed 95 regulation modes, among which seven genes (MMP1, TIMP1, TLR2, FCGR3A, IRF1, FAS, and TFF3) were hub molecules that are regulated at least by two of these five transcription factors simultaneously and were associated with hypoxia, inflammation, and immune disorder. Real-Time PCR and western blot showed increasing of HIF-1α in mRNA and protein levels as well as TIMP1, TFF3 in mRNA levels in gastric cancer tissues. The data are the first study to demonstrate HIF-1α-regulated transcription factors and their corresponding network genes in gastric cancer. Further study with a larger sample size and more functional experiments is needed to confirm these data and then translate into clinical biomarker discovery and treatment strategy for gastric cancer.

## Introduction

Gastric cancer is the fourth most common cancer and the second leading cause of cancer-related death in the world, which affects approximately 800,000 people and 65,000 cancer-related deaths annually [1]. Previous studies showed that aberrant cellular metabolism is a key feature during tumorigenesis and cancer progression [2], [3]. Specially, reprogramming of energy metabolism has been included as an emerging hallmark of cancer [4] and abnormal energy metabolism is detectable in different human cancer, i.e., cancer cells will reprogram their metabolism by increase in glycolysis instead of the mitochondrial oxidative phosphorylation to generate cell energy [5]. Tissue hypoxia is a crucial driving force leading to cell metabolism reprograming [6]. Under hypoxia environment, cell glycolysis is induced and leads to increase cell proliferation and in turn, forming a vicious cycle of hypoxia-proliferation-increasing hypoxia that promote cell transformation and cancer progression [7]. At the gene level, hypoxia-inducible factor-1 (HIF-1) is the primary oxygen-sensitive transcriptional activator and helps cells to adapt the low oxygen tension (hypoxia) [8]. HIF-1 is composed of a constitutively expressed β-subunit and a hypoxia-inducible α-subunit. The latter (HIF-1α) is only stabilized under hypoxic conditions and regulates HIF-1 transcriptional activity [9]. To date, HIF-1α is shown to activate multiple target genes that involve in crucial aspects of cancer biology, including erythropoiesis, angiogenesis, glucose metabolism, cell proliferation/survival and apoptosis [10]. HIF-1α can interact with various other cancer-related transcription factors (TFs) and form a complex TF-gene transcription regulatory network during cancer development and progression. Thus, a conception is not surprisingly raised that cancer cells have differential and pathological transcriptional patterns compared with normal cells [11]. Previous studies showed up-regulation of HIF-1α expression in gastric cancer tissues and cells [12], [13], whereas the precisely underlying regulatory mechanisms remain to be defined. Thus, in this study, we utilized the Affymatrix Exon Arrays to identify the differential gene expression profile in gastric cancer tissues, and performed real time PCR and western blot analyses to validate the data. We further constructed the aberrant TF-gene transcription regulatory network associated with HIF-1α expression by integration of transcriptional regulatory element database (TRED) [14] and gene expression profile using cytoscape software. This study could identify a systematic exposition of the associated transcriptional regulation modes related with hypoxia and provide insightful information for future biomarker discovery and novel treatment strategy for gastric cancer.

## Results and Discussion

### Profiling of differentially expressed genes in gastric cancer versus normal tissues

**Figure 1:**
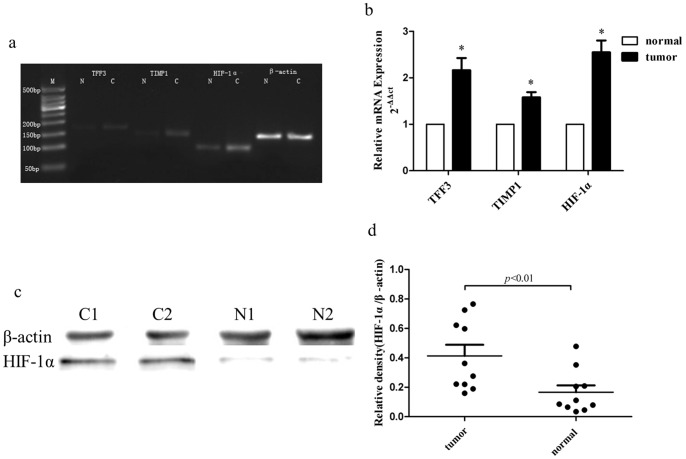
Validation of overexpression of HIF-1α, TIMP1 and TFF3 in 10 pairs of gastric cancer vs. normal tissues. a and b, Detection of HIF-1α, TIMP1 and TFF3 mRNA expression in gastric cancer vs. normal tissues using PCR and qRT-PCR. Levels of HIF-1α, TIMP1, TFF3 mRNA were 2.55±0.56, 1.58±0.25, 2.16±0.59 folds up-regulated in tumor tissues, respectively compared to those of the normal ones. *p<0.01. c and d, Western blot analysis of HIF-1α protein. Tumor tissues expressed higher level of HIF-1α protein compared to the normal ones [p<0.01 (d). N, normal tissues; C, cancer tissues (c)].

To identify the differentially expressed genes in gastric cancer, we utilized the Affymatrix Exon Arrays that contain 17,800 human genes to profile five pairs of gastric cancer and normal tissues (patients' information were showed in Table S1). We found a total of 2546 differentially expressed genes, of which 2422 were up-regulated and 124 were down-regulated (Table S2). Specifically, HIF-1α was significantly highly expressed in gastric cancer tissues compared to the adjacent normal tissues (P<0.01). We further validated the microarray data by performing quantitative real-time RT-PCR and western blot in another 10 pairs of gastric cancer vs. normal tissues (patients' information were showed in Table S1). The HIF-1α mRNA expression showed 2.55±0.56 fold up-regulation in tumor tissues vs. normal ones (p<0.01); western blot analysis showed a clear separation between the relative protein density of HIF-1α in cancer tissues (0.41±0.24) vs. normal ones (0.17±0.15) with p<0.01, results can be seen in Figure 1 and Figure S1. Indeed, a previous study showed that HIF-1α was ubiquitously expressed in human and mouse tissues under hypoxia [15] and in gastric cancer tissues [12], [13], overexpression of which was associated with poor prognosis of gastric cancer patients [12], [13]. Thus, we further analyzed HIF-1α overexpression-associated TFs and their potential targeting genes in gastric cancer tissues.

### Identification of HIF-1α overexpression-associated TFs and their potential targeting genes in gastric cancer tissues

**Figure 2:**
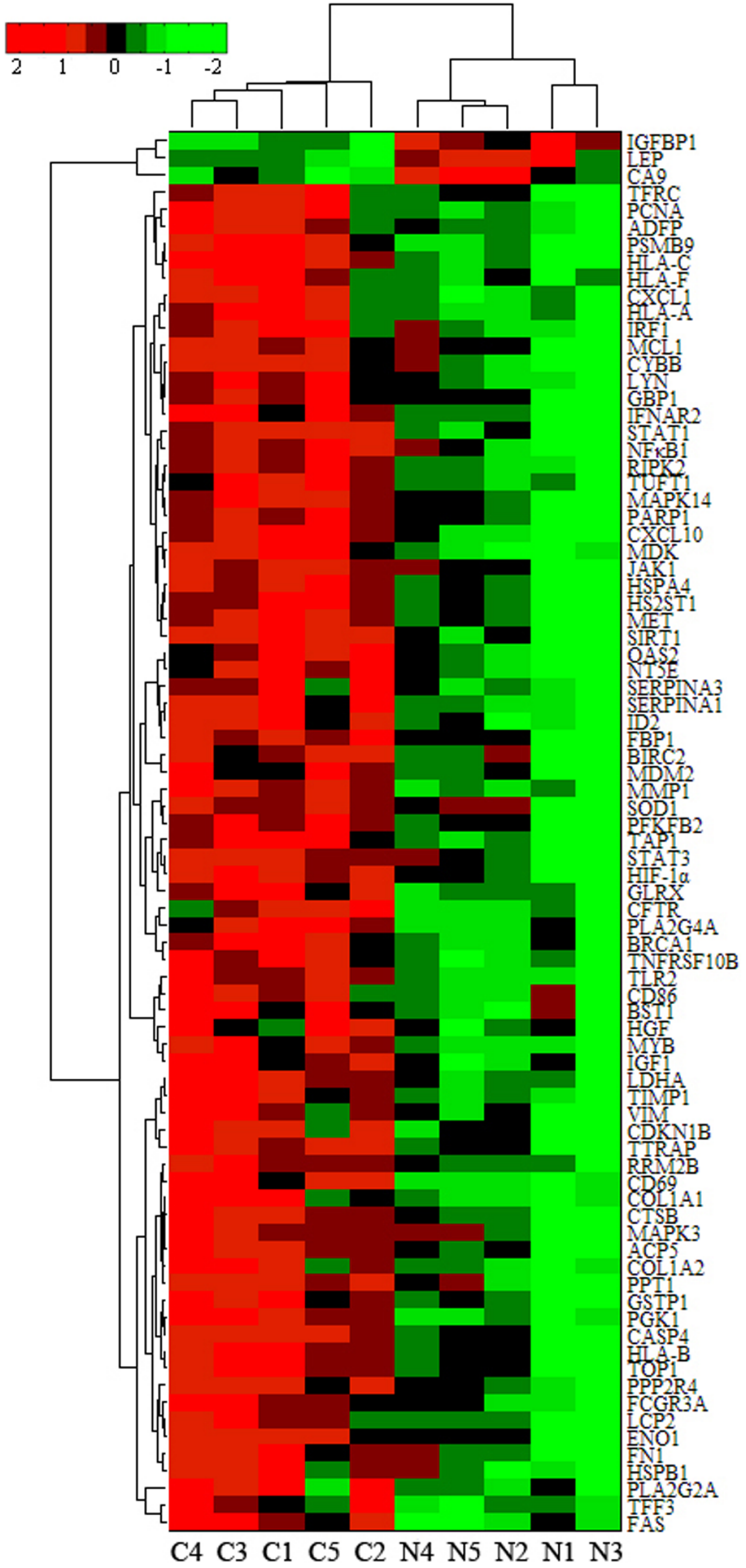
The bi-clusters analysis of these 82 differentially expressed genes in TF-gene regulatory network. Each row represents a gene and each column represents a sample, the “C” columns at the bottom represent cancer tissues, “N” columns represent normal tissues. >1 Red for high expression in cancer compared to normal and <1 green for low expression in cancer compared to normal ones.

To identify HIF-1α overexpression-associated TFs and their potential targeting genes, transcriptional regulatory element database (TRED) provides a unique tool to analyze both *cis*- and *trans*- regulatory elements in mammals, which helps to better understand the comprehensive gene regulations and regulatory networks, especially at the level of transcriptional regulations. Thus, using the integration gene expression profile and regulatory information from TRED, we analyzed HIF-1α and other four HIF-1α-related transcription factors (i.e., NFκB1, BRCA1, STAT3, and STAT1) that were all up-regulated in gastric cancer tissues and found that they formed these TF-gene regulatory networks with 82 genes, 79 of which were up-regulated and 3 were down-regulated (Table S3). Figure 2 showed the bi-clusters analysis of these 82 differentially expressed genes in gastric cancer tissues versus normal tissues.

After that, the Database for Annotation, Visualization and Integrated Discovery (DAVID) [16] was applied for functional annotation of these 82 differentially expressed genes. We listed the top four disease classes that associated with these 82 aberrant genes (Table 1) and found that the most significant class is Cancer with 29 genes followed by Infection (18 genes), Cardiovascular (25 genes) and Immune disease (26 genes).

**Table 1:** GENETIC_ASSOCIATION_DB_DISEASE_CLASS analysis of 82 genes in TF-gene regulatory network.

Term	P-Value	Fold enrichment	Benjamini	Genes
**Cancer**	2.53E-06	2.30	4.55E-05	TLR2, RRM2B, MDK, MMP1, TIMP1, TAP1, SERPINA1, FAS, FCGR3A, FN1, HLA-A, IGF1, CFTR, HLA-C, HLA-B, HGF, SOD1, BRCA1, CDKN1B, TFRC, PLA2G2A, IRF1, PCNA, MDM2, COL1A1, CTSB, PGK1, PARP1, GSTP1
**Infection**	4.82E-06	3.59	4.34E-05	TLR2, HLA-A, CFTR, HLA-C, OAS2, HLA-B, STAT1, MMP1, PSMB9, IFNAR2, TFRC, TAP1, IRF1, JAK1, FAS,SERPINA1, FCGR3A, GSTP1
**Cardiovascular**	4.77E-05	2.24	2.15E-04	TLR2, MMP1, TIMP1, TAP1, SERPINA3, SERPINA1, FAS, FN1,HSPA4, MYB, FCGR3A, HLA-A, IGF1, HLA-C, CFTR, HGF, HLA-B, STAT3, PSMB9, CDKN1B, PLA2G2A, COL1A2, MDM2, COL1A1, GSTP1
**Immune**	2.13E-04	1.99	7.66E-04	TLR2, OAS2, MMP1, TIMP1, CXCL10, TAP1, SERPINA3, SERPINA1, FAS, FCGR3A, HLA-A, IGF1, CFTR, HLA-C, HLA-B, STAT3, PSMB9, IFNAR2, CYBB, CD86, CTSB, IRF1, TNFRSF10B, COL1A1, PARP1, GSTP1

### Identification of gastric cancer-related transcription factor-gene (TF-gene) network

**Figure 3:**
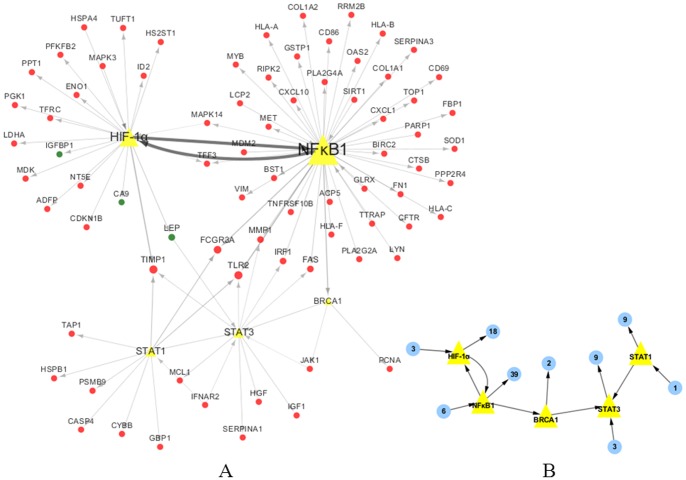
TF-gene network of these 82 differentially expressed genes in gastric cancer tissues. Red circles in A are up-regulated genes, whereas green circles are down-regulated genes and the yellow triangles are these five key TFs. B, The brief framework of this network. The circles are the clustered genes and the number of genes is shown inside. The direction of the arrow is from the Source to the Target.

Based on transcriptional regulatory element database and gene expression profile, we constructed the transcriptional regulatory network related to HIF-1α ↔ NFκB1 → BRCA1 → STAT3 ← STAT1 with these 82 genes in gastric cancer tissues. Our data showed that these 82 genes can form 95 different regulation modes (Figure 3A) and the detailed TF-gene regulation modes information is listed in Table S4.

In order to better understand the regulatory network, we built a brief framework of the network (Figure 3B). Transcription factors HIF-1α ↔ NFκB1 → BRCA1 → STAT3 ← STAT1 were able to form the framework of the regulatory network by which directly regulated 21, 45, 2, 12, and 10 genes, respectively. NFκB1 was directly regulated by HIF-1α and it was true that the majority of the regulatory network were directly regulated by HIF-1α (21/82) and NFκB1 (45/82), the key regulators linked with hypoxia and inflammation in cancers [17]. Gastric cancer is characterized by tissue hypoxia and chronic inflammation (such as *Helicobacter* pylori infection). In our current study, HIF-1α was significantly up-regulated in gastric cancer compared to the adjacent normal tissues (P<0.01). Moreover, our current data showed that expression of more than 20 genes that are directly regulated by HIF-1α was altered in gastric cancer tissues, including NFκB1, the key regulator molecule in inflammation and cancer [18] and targeting of NFκB could be useful in chemoprevention of various human cancers [19].

The downstream of the regulatory pathway network is mainly regulated by STAT3 (12/82) and STAT1 (10/82), members of signal transducer and activator of transcription family (STATs). STATs signaling with Jak is a canonical pathway to regulate genes that are involved in many physiological processes by transferring signals from the cell membrane to the nucleus [20]. To regulate paracrine cytokine signaling and alterations in metastatic sites, STAT3 exerts both tumor-intrinsic and extrinsic effects [21]. Targeting Jak-STAT3 signaling pathway is considered as a potential therapeutic strategy, especially in the context of tumor inflammation and immunity [21]. Continuous deregulation of genes by persistently activated NFκB and STAT3 in tumor microenvironment is two crucial aspects for inflammation and malignant progression [17]. A previous study showed a cooperative effect of STAT3 and HIF-1α on activation of genes under hypoxia environment in renal cell carcinoma cells [22]. The specific mechanism of Jak-STAT activation, especially STAT3 in gastric cancer remains to be determined, although our current data showed significantly higher level of JAK1, STAT3 and STAT1 expression in gastric cancer tissues.

### Function analysis of the hub-genes

**Figure 4:**
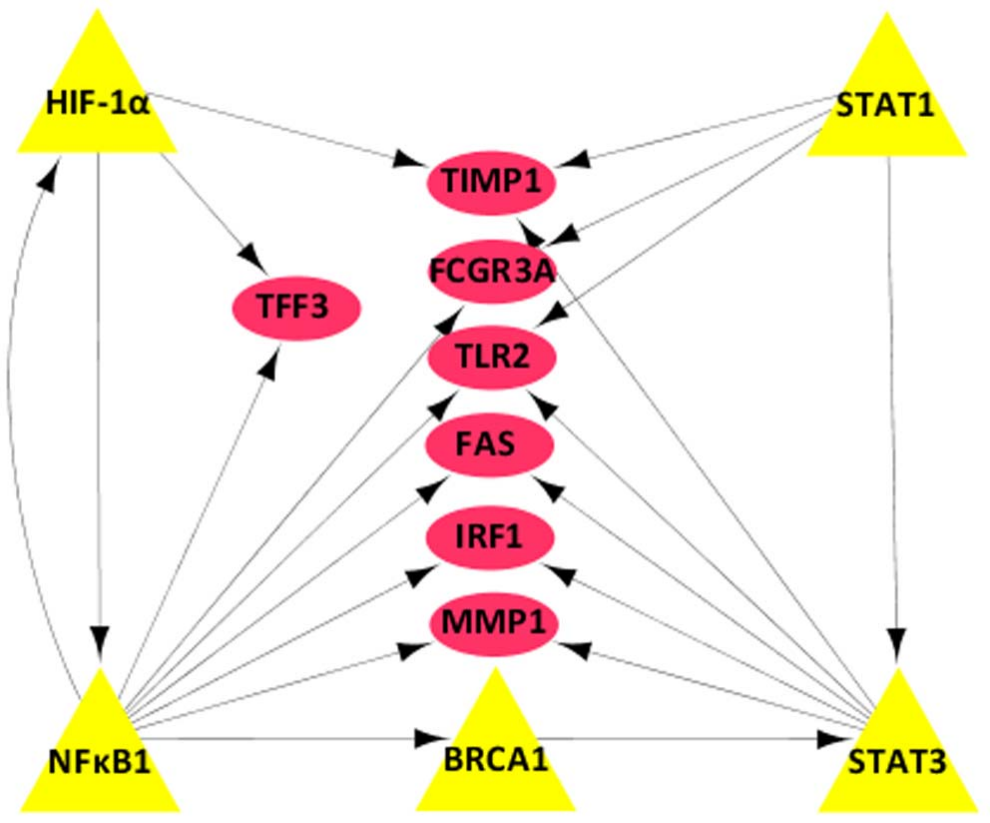
The hub genes are regulated by at least two TFs in this TF-gene regulatory network. Ellipses are hub genes that are regulated by transcription factors, the triangles are these five transcription factors in the TF-gene regulatory network.

A given transcription factor may regulate dozens, if not hundreds, of the target genes, while one gene could be regulated by several different TFs in gene regulatory networks. Thus, we assumed that hub genes being regulated by several transcription factors simultaneously in gastric cancer, which may have synergistic effects on human carcinogenesis. In the current study, we identified seven genes (including MMP1, TIMP1, TLR2, FCGR3A, IRF1, FAS, and TFF3) that can be directly regulated by at least two key transcription factors, most of them are hub nodes that linking with NFκB1 and STATs pathway (Figure 4). Since transcription factors regulate the target genes through a transcription-depended manner to modulate their mRNA expression, here we performed qRT-PCR to examine expression of TIMP1 and TFF3 mRNA, two target genes of HIF-α The relative expression of TIMP1 and TFF3 mRNA was 1.58±0.25 and 2.16±0.59 fold up-regulated in ten tumor vs. normal tissues, respectively (Figure 1).

In addition, the family of matrix metalloproteinases (MMPs) is the main extracellular matrix remodeling enzymes, activity of which is the result of interaction between tumor cells and tumor microenvironment and is tightly controlled by transcriptional activation, including a complex proteolytic activation cascade as well as endogenous system of tissue inhibitors of metalloproteinases (TIMPs) [23]. MMP1 has been reported to be involved in gastric cancer cell invasion [24]. Moreover, TLR2 is member of toll-like receptors and plays a fundamental role in pathogen recognition and activation of innate immunity by activation of NFκB. TLR2 may function as an initiator for giving the infected or injured cells a second chance to develop into cancer cells and uncontrolled cell proliferation [25]. Meanwhile, the Fc fragment of IgG, low affinity IIIa receptor (FCGR3A, also known as CD16a) belongs to the Fc gamma receptor family (FCGR). *FCGR3A* polymorphism was associated with susceptibility to certain autoimmune diseases and FCGR3A has an important role in removing the immune complexes from the body and also participates in cytotoxic responses against tumor cells and infectious agents [26]. The interferon regulatory factor (IRF)-1 is also an immune active molecule and inflammatory process regulator, the activation of IRF-1 and NF-κB was found to be concurrently activated in melanoma [27]. In addition, polymorphisms of the trefoil factor 3(*TFF3*) promoter were associated with gastric cancer susceptibility [28] and TFF3 was regulated by both HIF-1 and NFκB [29]. Overexpression of TFF3 was an independent indicator for overall survival of gastric cancer patients [30]. Again, FAS (also known as TNFSF6/CD95/APO-1) belongs to tumor necrosis factor receptor superfamily (member 6) and plays an essential role in regulation of extrinsic apoptosis pathway [31]. Reduced FAS expression was associated with the increased risk of cancer by downregulation of FAS-mediated apoptosis [32]. However, our current data showed a contradictory high expression level of FAS in gastric cancer tissues ad further study is needed to confirm it. Overall, altered expression of these genes in gastric cancer tissues needs further verification as biomarkers for gastric cancer diagnosis and prognosis. These genes are crucial in inflammation and immune related disease, which may further indicate the importance of *Helicobacter* pylori infection in gastric cancer development and progression.

## Materials and Methods

### Tissue specimens

A total of 15 gastric cancer patients were recruited for cancer and the distant normal tissue collection from The First Hospital of Jilin University, Changchun, China. This study was approved by the Ethics Committee of College of Basic Medical Sciences, Jilin University, each patient was consented in a written informed consent form. The data were analyzed anonymously. All tissues were taken from surgery room and snap-frozen and stored in liquid nitrogen within 10 min after the resection. The TNM and histological classification were performed according to World Health Organization (WHO) criteria.

### RNA isolation and microarray hybridization and scanning

Tissue RNA was isolated using Trizol (Invitrogen, CA, USA) and further purified using the RNeasy Mini kit (Qiagen, Düsseldorf, Germany) according to the manufacturer's instructions. RNA concentration was then determined using the UV2800 ultraviolet spectrophotometer (UNIC, NY, USA) with A260/A280 ratio between 1.8∼2.0 and RNA concentration was ranged from 100 ng/µl to 1 µg/µl.

GeneChip Human Exon 1.0 ST (Affymetrix, CA, USA) was utilized to profile differentially expressed genes in gastric cancer tissues vs. the normal ones according to the protocol provided by Affymetrix (P/N 900223). Briefly, 1 µg RNA template was used to reversely transcribed into cDNA and cDNA samples were digested into cDNA fragments with endonucleases and then labeled with the DNA labeling reagent provided by Affymetrix. After that, the labeled cDNA samples were used as probes to hybridize to the array chips by incubation at 45°C and rotated at 60 rpm for 17 h. After washed and stained the chips after hybridization, the chips were scanned using GeneChip Scanner3000 with GeneChip Operating Software (GCOS). All instruments, chips, and reagents were all purchased from Affymetrix.

### Analysis of differentially expressed genes in cancer versus normal tissues

GeneChip Operating Software was applied to analyze the chips and extract the raw images signal data. The GEO DataSets of NCBI accession number of our study is: GSE56807. Raw signal data were then imported and analyzed with Limma algorithm to identify the differentially expressed genes. The linear models and empirical Bayes methods were to analyze the data. This prevented a gene with a very small fold change from being judged as differentially expressed just because of an accidentally small residual SD. The resulting P values were adjusted using the BH FDR algorithm. Genes were considered to be significantly differentially expressed if both the FDR values was <0.05(controlling the expected FDR to no more than 5%) and gene expression showed at least 2-fold changes between cancer and their corresponding normal tissues with Log2FC > 1 or log2FC < -1, P-value < 0.05.

### Quantitative real-time RT-PCR

For qRT-PCR analysis, less than 5 µg total RNA was reverse transcribed to cDNA with 1^st^ strand cDNA Synthsis Kit (Takara, Dalian, China); the expression of mRNA for human HIF-1α, TIMP1 and TFF3 were examined by qRT-PCR with SYBR Premix Ex Taq (Takara, Dalian, China) and Applied Biosystems 7300 Fast Real-Time PCR System. The relative expression of mRNA were normalized to β-actin expression by comparative Ct method (2^−ΔΔCt^,ΔCt  =  Ct _target_-Ct _β-actin_, ΔΔCt  =  ΔCt_tumor_-ΔCt_normal_). All primers were designed with Primer Premier 6 Software, primer sequences for amplification were listed in Table 2. Data from qRT-PCR were analyzed with GraphPad Prism Version 5.0, differences between groups were statistically evaluated by sample one-tailed Student's t-test with p value <0.05 considered as significant.

**Table 2:** Primer sequences used for qRT-PCR amplification.

Genes	Forward primers	Reverse primers
HIF-1α	5′-TAGCCGAGGAAGAACTATGAAC-3′	5′CTGAGGTTGGTTACTGTTGGTA-3′
TIMP1	5′-CTGTTGTTGCTGTGGCTGATA-3′	5′-ACGCTGGTATAAGGTGGTCTG-3′
TFF3	5′-AATGCACCTTCTGAGGCACCT-3′	5′-CGTTAAGACATCAGGCTCCAGAT-3′
β-actin	5′-CTGGAACGGTGAAGGTGACA-3′	5′-AAGGGACTTCCTGTAACAATGCA-3′

### Western blot analysis

About 1 mm^3^ of tissue samples were polished with liquid nitrogen then homogenized in cell lysis buffer (Beyotime, China) in 4°C for 30 min, removed cell debris by centrifuging at 10000 rpm for 20 min in 4°C. The protein concentration was analyzed by Bradford protein assay (Bio-Rad, USA). The whole protein was separated with 10% SDS-PAGE and then transferred to a PVDF membrane (0.45 µm) for 2 h. After 2 h of blocking by 5% milk in TBST, incubated the membrane with mouse anti-HIF-1α (Santa Cruz, CA, USA) at 1∶200 dilution and mouse anti-β-actin (proteintech, USA) at 1∶2000 dilution in 4°C for 12 h and followed by 2 h incubating with goat anti-mouse IgG (proteintech, USA) at 1∶2000 dilution. After washing by TBST, detected the membrane signals using enhanced chemiluminescence ECL (Beyotime, China). The Image J software was applied for quantitative analysis of HIF-1α signal intensities with normalized with β-actin levels. Data were analyzed with GraphPad Prism Version 5.0, differences between groups were statistically evaluated by sample one-tailed Student's t-test with p value <0.05 considered as significant.

### Construction of transcription factor gene network based on gene expression profile and transcriptional regulatory element database

Transcription factor (TF) gene network was constructed based on gene expression profile and transcriptional regulatory element database (TRED) using cytoscape software according to the regulatory interaction and the differential expression values of each TF and gene. The adjacency matrix of TFs and genes was made by the attribute relationships among all genes and TFs. The ellipse in TF-gene network represented genes with red (up-regulated) and green (down-regulated), the triangles represents transcription factors. The relationship between TF and their targets were represented by arrows, direction of the arrow was from the Source to the Target.

### Analysis of disease associated genes and gene pathway annotation

Database for Annotation, Visualization and Integrated Discovery(DAVID) functional annotation software was applied to analyze the functional enrichment of aberrant genes. “GENETIC_ASSOCIATION_DB_DISEASE_CLASS” option provided the information about disease association enrichment of gene clusters. We selected “GENETIC_ASSOCIATION_DB_DISEASE_CLASS” for identifying disease class enrichment and “KEGG_PATHWAY” for pathway enrichment with Benjamini method determining the significant enrichment score≥1.3.

## Supporting Information

Figure S1 **Western blot analysis of HIF-1α in 10 pairs of gastric cancer and normal tissues.**
(DOC)

Table S1 **Patients data.**
(DOC)

Table S2 **Summary of 2546 differentially expressed genes in gastric cancer tissues compared to the distant normal tissues.** Gene expression levels in gastric cancer tissues vs. the distant normal tissues were at least 2-fold different with a p-value <0.05.
(XLSX)

Table S3 **Summary of these82 differentially expressed genes in the TF-regulatory network in gastric cancer tissues.**
(XLSX)

Table S4 **The 95 regulation modes formed by 82 differential genes in TF-gene regulatory network.** All regulation information was derived from transcriptional regulatory element database (TRED).
(XLSX)
