# Supplementary figures and images for: Altered Expression of Hypoxia-Inducible Factor-1α (HIF-1α) and Its Regulatory Genes in Gastric Cancer Tissues
Source: PLoS One. 2014 Jun 13;9(6):e99835. doi: 10.1371/journal.pone.0099835 (PMC4057318; doi:10.1371/journal.pone.0099835)

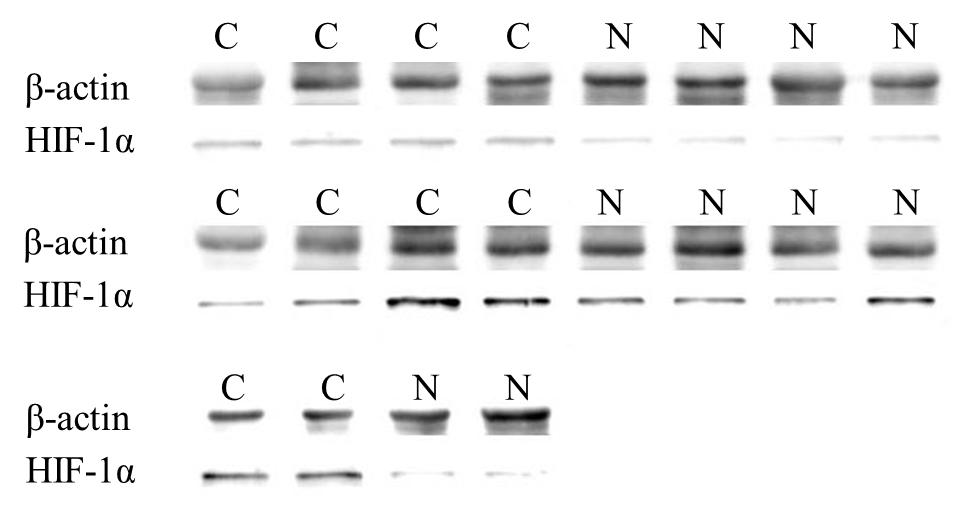


Figure S1 Western blot analysis of HIF-1α in 10 paired of gastric cancer and normal tissues

Supplement: Figure S1 — Western blot analysis of HIF-1α in 10 pairs of gastric cancer and normal tissues. (DOC) [file pone.0099835.s001.doc]
