# Supplementary material for: Altered Expression of Hypoxia-Inducible Factor-1α (HIF-1α) and Its Regulatory Genes in Gastric Cancer Tissues
Source: PLoS One. 2014 Jun 13;9(6):e99835. doi: 10.1371/journal.pone.0099835 (PMC4057318; doi:10.1371/journal.pone.0099835)
Supplement: Table S1 — Patients data. (DOC) [file pone.0099835.s002.doc]

Table S1. Clinicopathological characteristics of the study samples

| Case | Participate | Clinical parameters | | | |
| --- | --- | --- | --- | --- | --- |
|  |  | ***Gender*** | ***Age*** | ***Differentiation status*** | ***Clinical stage*** |
| 1 | microarray | Female | 46 | Poorly differentiated | IV |
| 2 | microarray | Male | 55 | Moderately differentiated | III |
| 3 | microarray | Female | 49 | Moderately differentiated | III |
| 4 | microarray | Female | 69 | Highly differentiated | II |
| 5 | microarray | Female | 63 | Highly differentiated | I |
| 6 | RT-qPCR and western | Male | 47 | Poorly differentiated | III |
| 7 | RT-qPCR and western | Male | 71 | Moderately differentiated | III |
| 8 | RT-qPCR and western | Female | 65 | Poorly differentiated | IV |
| 9 | RT-qPCR and western | Male | 66 | Highly differentiated | II |
| 10 | RT-qPCR and western | Female | 57 | Moderately differentiated | III |
| 11 | RT-qPCR and western | Female | 54 | Highly differentiated | II |
| 12 | RT-qPCR and western | Male | 60 | Poorly differentiated | IV |
| 13 | RT-qPCR and western | Female | 63 | Moderately differentiated | III |
| 14 | RT-qPCR and western | Male | 72 | Moderately differentiated | III |
| 15 | RT-qPCR and western | Male | 50 | Highly differentiated | II |
